# The effectiveness of M-health technologies for improving health and health services: a systematic review protocol

**DOI:** 10.1186/1756-0500-3-250

**Published:** 2010-10-06

**Authors:** Caroline Free, Gemma Phillips, Lambert Felix, Leandro Galli, Vikram Patel, Philip Edwards

**Affiliations:** 1 Department of Nutrition and Public Health Intervention Research, Faculty of Epidemiology and Population Health, London School of Hygiene and Tropical Medicine WC1E 7HT UK

## Abstract

**Background:**

The application of mobile computing and communication technology is rapidly expanding in the fields of health care and public health. This systematic review will summarise the evidence for the effectiveness of mobile technology interventions for improving health and health service outcomes (M-health) around the world.

**Findings:**

To be included in the review interventions must aim to improve or promote health or health service use and quality, employing any mobile computing and communication technology. This includes: (1) interventions designed to improve diagnosis, investigation, treatment, monitoring and management of disease; (2) interventions to deliver treatment or disease management programmes to patients, health promotion interventions, and interventions designed to improve treatment compliance; and (3) interventions to improve health care processes e.g. appointment attendance, result notification, vaccination reminders.

A comprehensive, electronic search strategy will be used to identify controlled studies, published since 1990, and indexed in MEDLINE, EMBASE, PsycINFO, Global Health, Web of Science, the Cochrane Library, or the UK NHS Health Technology Assessment database. The search strategy will include terms (and synonyms) for the following mobile electronic devices (MEDs) and a range of compatible media: mobile phone; personal digital assistant (PDA); handheld computer (e.g. tablet PC); PDA phone (e.g. BlackBerry, Palm Pilot); Smartphone; enterprise digital assistant; portable media player (i.e. MP3 or MP4 player); handheld video game console. No terms for health or health service outcomes will be included, to ensure that all applications of mobile technology in public health and health services are identified. Bibliographies of primary studies and review articles meeting the inclusion criteria will be searched manually to identify further eligible studies. Data on objective and self-reported outcomes and study quality will be independently extracted by two review authors. Where there are sufficient numbers of similar interventions, we will calculate and report pooled risk ratios or standardised mean differences using meta-analysis.

**Discussion:**

This systematic review will provide recommendations on the use of mobile computing and communication technology in health care and public health and will guide future work on intervention development and primary research in this field.

## Background

M-health, the use of mobile computing and communication technologies in health care and public health, is a rapidly expanding area of research and practice. M-health programmes and interventions use mobile electronic devices (MEDs), such as personal digital assistants (PDAs) and mobile phones, for a range of functions from clinical decision support systems and data collection tools for healthcare professionals[1,2], to supporting health behaviour change and chronic disease management by patients in the community[3].

Current documented M-health interventions and programmes include: mobile phone text messaging to support management of diabetes, hypertension, asthma, eating disorders and HIV treatment[3-7]; mobile phone text messaging and PDAs as aids to smoking cessation, body weight loss, reducing alcohol consumption, sexually transmitted infection prevention and testing[3,5-7]; PDAs for data collection in healthcare and health research[1,8-10] and to support medical education and clinical practice[2,11-13]. Whilst the majority of M-health interventions are reported from high income countries, there is an emerging literature on the application of mobile technologies in low income countries[1,7,14-16].

Mobile communication technology is the fastest growing sector of the communications industry in low income countries[17,18]. In the last two decades, the global digital divide has narrowed most for mobile phone use, with many low income countries "leap-frogging" over fixed-line communications technologies, straight to expansion of wireless cellular communication networks[18-20]. Whilst wireless communication network coverage and mobile phone ownership are not universal, or equally distributed, in low income countries, and the technology accessible to most of the population lags far behind that available in high income countries[19,21], there is still huge potential for M-health interventions and programmes to have positive effects on health outcomes in resource-poor settings[15].

Mobile technologies have a number of key features that give them an advantage over other information and communication technologies in particular activities within health care and public health. Firstly, many MEDs have wireless cellular communication capability, providing the potential for continuous, interactive communication from any location e.g. telephone calls, text and multimedia messaging and also internet access via Wireless Application Protocol (WAP) or mobile broadband internet. Secondly, the devices are portable because of their small size, low weight and rechargeable, long-life battery power. Finally, many MEDs have sufficient computing power to support multimedia software applications. The combination of these features varies between specific devices and their relative importance will change with the health activity in which they are used. However, with advances in technology development, single devices increasingly possess many or all of these functions.

Existing systematic reviews of M-health interventions, and recently published protocols[22-26], focus on the application of specific devices (e.g. mobile phones[6,9,10,13,27]) or specific functions (e.g. text messaging[3,4,28]) to individual diseases or healthcare fields (e.g. diabetes care or chronic disease management[5,10,28]). In this systematic review, we propose to look all types of mobile technologies and all health outcomes, to provide a broad overview of the M-health sector at this relatively early point in its development. This will allow us to describe the use of different mobile technology functions across a range of healthcare and public health fields, from health behaviour to clinical outcomes such as medication compliance and service use, both to highlight similarities in mechanisms of action for a particular device or function and to suggest where they may be usefully transferred to new areas. Finally, we will identify studies of newer technologies such as Smartphones and portable media players, which are unlikely to be captured in technology-specific reviews.

The objectives of this review are to: (1) describe the uses of mobile computing and communication technologies by patients, healthcare professionals and the general public in the context of health service and public health interventions that have been evaluated in controlled studies; (2) assess the effectiveness of mobile technologies for improving health and health service outcomes in high, middle and low income countries; and (3) describe the acceptability of mobile technologies to patients, healthcare professionals and the general public, in the context of health service and health promotion interventions.

## Methods and Design

### Review Inclusion Criteria

#### Types of technology

For the purposes of this review, MEDs will be defined as devices which either have interactive wireless cellular communication capability and/or those which run software applications and are highly portable. We will therefore include interventions using: mobile phones; personal digital assistants (PDA) and PDA phones (e.g. BlackBerry, Palm Pilot); Smartphones (e.g. the iphone); Enterprise digital assistants (EDA); portable media players (i.e. MP3-players and MP4-players, e.g. ipod); handheld video-game consoles (e.g. Playstation Portable (PSP), Nintendo DS); handheld and ultra-portable computers such as tablet PCs (e.g. the ipad) and Smartbooks. A summary of the functions available with each of these devices is provided in Table 1. Desktop personal computers, notebook (laptop) computers, subnotebook computers, netbooks, pagers, handheld calculators and pedometers are not considered to be MEDs and interventions delivered exclusively on these devices will be excluded. Whilst notebook computers are designed to be portable we have excluded them from this review because, in comparison to MEDs as defined above, notebook computers: (1) have substantially shorter battery life; (2) weigh substantially more; (3) do not provide quick access to information and programmes (PDAs and mobile phones have "always on" functionality); and (4) are not suitable for healthcare activities that require the user to be standing or mobile.

**Table 1:** Functions of mobile electronic devices included in the review

	Mode of communication								
	Voice	SMS	MMS	Email	WAP internet	Wireless cellular broadband	Audio	Video	Custom/additional software support
Mobile phone									
Basic model ^1^									
(e.g. Nokia 1280)^1^	✓	✓							
High-end model^1^									
(e.g. Nokia 6303i)^1^	✓	✓	✓	✓	✓		✓	✓	
PDA	✓	✓	✓	✓		(✓)			✓
Smartphone	✓	✓	✓	✓		✓	✓	✓	
PDA phone	✓	✓		✓	✓				
Enterprise digital assistant									✓
Portable media player							✓	✓	
Handheld video games console							✓	✓	
									

Brackets indicate where functions are available on only some higher specification models.

Abbreviations: MMS, multimedia messaging service; PDA, personal digital assistant; SMS, short messaging service; WAP, wireless application protocol.

^1 ^Source GSM Arena (http://www.gsmarena.com/, accessed 09.06.10)

#### Types of intervention

Interventions must aim to improve or promote health or health service use and quality, employing any MED. This includes: (1) interventions designed to improve diagnosis, investigation, treatment, monitoring and management of disease; (2) interventions to deliver treatment or disease management programmes to patients, health promotion interventions, and interventions designed to improve treatment compliance; and (3) interventions to improve health care processes e.g. appointment attendance, result notification, vaccination reminders. We will therefore include:

- Any intervention delivered using an MED owned or directly used by a patient or lay person;

- Any clinical or practice aid delivered using an MED owned or directly used by a healthcare professional;

- Any data collection or storage for the purposes of healthcare or health research using an MED.

**Figure 1:**
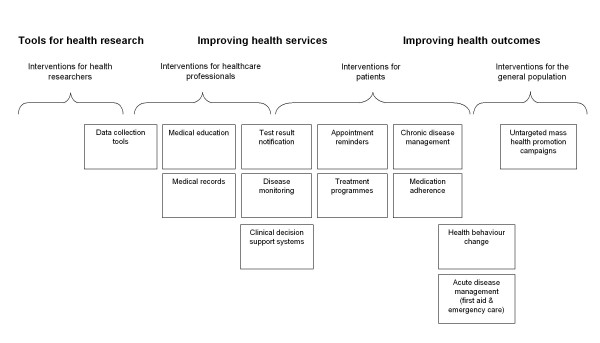
**Conceptual framework for mobile electronic device intervention classification**.

We anticipate that interventions will aim to address one of the health domains shown in Figure 1, although it is likely that further MED applications will be identified in the review and this framework will be updated.

#### Types of studies

Studies must have used a controlled design to evaluate an MED intervention. We will include both randomised controlled trials and studies with non-random treatment group allocation. Previous reviews have highlighted the difficulty in assessing the impact of MED on health outcomes when the MED is used as an adjunct to other interventions and services e.g. text messaging in addition to clinician appointments for managing hypertension[4]. We will therefore only include studies where the MED is the primary intervention component under evaluation. We will include studies evaluating:

- Interventions delivered via a single MED to the treatment group (N.B. this could therefore include an intervention where a number of behaviour change techniques are used, e.g. prompts, reminders and patient-initiated support, but all delivered through a single MED, e.g. SMS to and from a mobile phone), where the control group receives no MED intervention;

- Multi-MED interventions where the treatment group receives one or more interventions delivered through multiple MED, but no interventions through other (non-MED) modes and the control group receives no MED interventions;

- Mixed MED and non-MED interventions where the treatment and control group both receive all non-MED components of the intervention and the MED intervention is delivered only to the treatment group, e.g. SMS plus group counselling for smoking cessation in the treatment group and the control group receives group counselling only;

- Interventions delivered by different MED e.g. iphone vs. regular mobile phone;

- The features and components of interventions delivered on a particular MED, such as the intensity, personalisation, content, duration and timing of an intervention, and the degree of software or other component customisation.

We will exclude studies evaluating:

- Mixed MED and non-MED interventions where the treatment and control group both receive the MED component;

- Interventions where there are other treatment differences between the treatment and control groups besides the delivery of the MED component(s).

#### Types of participants

There will be no limits on study participants in terms of age, gender, ethnicity, morbidities (for patients and the general population) or staff role and occupation (for healthcare professionals e.g. nurse, surgeon, or physiotherapist). There will be no limits on study setting and we will include studies at all levels of healthcare setting (i.e. primary, secondary and tertiary healthcare) and those conducted in the community.

#### Types of outcome measures

All outcome measures reported in studies meeting the inclusion criteria will be extracted, both objective and self-reported measures. User-acceptability will be assessed as a self-reported outcome for all intervention types. We will also seek data on unintended adverse consequences of the interventions and process outcomes (e.g. repetitive strain injury or involvement in road traffic accidents).

Primary outcome measures will include any objective measure of health, or health service delivery or use. These may include biologically confirmed smoking cessation or mean change in body mass index, blood pressure, blood lipids or blood glucose levels. Secondary outcome measures will include: cognitive outcomes relating to knowledge, motivation, self-efficacy and intention; self-reported outcomes related to health behaviours (e.g. number of cigarettes smoked), chronic disease management, health service delivery or use. Examples of outcome measures for each health domain in Figure 1 are provided in Table 2.

**Table 2:** Example outcome measures for anticipated MED-based interventions shown in Figure 1

MED-based intervention	Example outcome measures	
	Objective outcomes	Self-reported outcomes
Clinical decision support systems - diagnosis	• Adherence to clinical protocol • Diagnosis of disease or disease risk (primary prevention) • Treatment e.g. medication prescribed	• Use of clinical protocol • Ease of clinical protocol use/comprehensibility
Clinical decision support systems - disease management	• Successful disease management e.g. diabetes control measured by HbA1C, peak flow • Medication prescribed • Investigations arranged • Health outcomes e.g, cardiac events, mortality, diarrhoea, breast feeding	• Use of clinical protocol • Ease of clinical protocol use/comprehensibility
Medical education	•Changes in clinical knowledge/skills	• Ease of use
Disease monitoring	• Data collected • Disease control e.g. HbA1c, peak flow • Timeliness of data collection	• Ease of data collection
Data collection tools Medical records	• Data completeness • Timeliness of data collection	• Ease of data collection • Ease of diagnosis
Test result notification	• Time to result notification • Time to treatment initiation	• Patient satisfaction with clinic service • Clinician preference for mode of notification
Appointment reminders	• Percent of appointments missed • Percentage of appointments cancelled in advance	• Patient intention to attend appointment
Treatment programmes	• Treatment outcomes e.g. change in depression score	• Patient satisfaction with treatment • Perceived level of support • Perceived changes in health status/disease condition
Chronic disease management Medication adherence	• Disease management e.g. diabetes control measured by HbA1C or asthma management expiration peak flow rate • Percent of medication doses taken on time	• Self-efficacy to manage condition/medication
Health behaviour change	• Health outcome e.g. body mass index (weight loss) or blood cotinine levels (smoking cessation)	• Self-efficacy to increase exercise/control dietary intake • Self reported behaviour
Acute disease management (first aid/emergency care)	• Cardiopulmonary resuscitation chest compression rate/depth/accuracy/hand position	• Perceived clarity of instructions
Untargeted mass health promotion campaigns	• Coverage • Uptake of services or treatment	• Knowledge

### Literature search

We will use a three-part search strategy to identify studies meeting the inclusion criteria above that have been published since 1990: (1) we will search electronic bibliographic databases for published work, using a comprehensive search strategy for mobile technology interventions; (2) we will search trial registers for ongoing and recently completed trials; (3) we will search the reference lists of primary studies included in the review and the reference lists of relevant, previously published reviews.

#### Electronic bibliographic databases

The following electronic bibliographic databases will be searched: MEDLINE, EMBASE, PsycINFO, Global Health, The Cochrane Library (Cochrane Database of Systematic Reviews, Cochrane Central Register of Controlled Trials (CENTRAL), Cochrane Methodology Register), NHS Health Technology Assessment Database, and Web of Science (science and social science citation index). The search strategy will include only terms relating to or describing mobile technologies because we will include all types of intervention and health or health service outcomes meeting the inclusion criteria described above. The search strategy for MEDLINE is shown in Additional File 1. All of these terms will be combined with the Cochrane Library MEDLINE filter for controlled trials of interventions. The mobile technology search terms will be adapted for use with other bibliographic databases in combination with database-specific filters for controlled trials, where these are available. There will be no language restrictions. The searches will be re-run immediately prior to analysis and further studies retrieved for inclusion, to ensure that the most current information is presented in the review.

We will include data from dissertations that meet the inclusion criteria, where these are indexed in the above databases and retrieved in our search. We will not be retrieving or including any unpublished data.

#### Trial registers

Ongoing, recently completed and unpublished clinical trials meeting the inclusion criteria described above will be identified from the following research registers: National Institutes of Health clinical trials registry (US); National Institute for Health Research Clinical Research Network Portfolio Database (UK); National Research Register Projects Database Archive (UK); and Current Controlled Trials (includes the International Standard Randomised Controlled Trial Number Register).

### Study screening and selection

Titles and abstracts of studies retrieved using the search strategy and those from additional sources will be screened independently by two review authors to identify studies that potentially meet the inclusion criteria outlined above. The full text of these potentially eligible studies will be retrieved and independently assessed for eligibility by two review authors. Any disagreement between the two review authors over the eligibility of particular studies will be resolved through discussion with a third review author.

### Data extraction

A standardised, pre-piloted form will be used to extract data from the included studies for assessment of study quality and evidence synthesis. Extracted information will include: study setting (including country); study population and participant demographics and baseline characteristics; MED used; details of the intervention and control conditions; study methodology; recruitment and study completion rates; outcomes and times of measurement; indicators of acceptability to users; suggested mechanisms of intervention action; information for assessment of the risk of bias (see below). Two review authors will independently extract data, discrepancies will be identified and resolved through discussion (with a third author where necessary). Missing data will be requested from study authors via email.

### Assessing risk of bias

Two review authors will independently assess the risk of bias in included studies by considering the following characteristics, as recommended by the International Cochrane Collaboration[29]:

- Randomisation sequence generation: was the allocation sequence (used to assign participants to the treatment and control groups) adequately generated? (This criterion only applies to randomised controlled trials.)

- Treatment allocation concealment: was the allocated treatment adequately concealed from study participants and clinicians and other healthcare or research staff at the enrolment stage?

- Blinding: were the personnel assessing outcomes and analysing data sufficiently blinded to the intervention allocation throughout the trial?

- Completeness of outcome data: were participant exclusions, attrition and incomplete outcome data adequately addressed in the published report?

- Selective outcome reporting: is there evidence of selective outcome reporting and might this have affected the study results?

- Other sources of bias: was the trial apparently free of any other problems that could produce a high risk of bias?

Disagreements between the review authors over the risk of bias in particular studies will be resolved by discussion, with involvement of a third review author where necessary. The level of risk of bias in each of these domains will be presented separately for each study in tables in the final review publication.

### Analysis

#### Descriptive analysis

We will provide a narrative synthesis of the findings from the included studies. We will structure the narrative synthesis by describing the studies according to the following characteristics:

- The type of intervention e.g. individual behaviour change, chronic disease self-management, clinic appointment reminders or clinical diagnostic aid (as outlined in Figure 1);

- The type of MED used;

- The target population characteristics e.g. age, gender, ethnicity, socioeconomic status and/or education level, low/middle/high income country setting (classified according to the World Bank List of Economies[30]);

- The type of outcome e.g. smoking cessation or weight loss;

- Intervention content - features of the MED employed (e.g. SMS, video), intervention components such as reminders, feedback or peer support, intensity, duration, personalisation and theoretical basis (if stated).

We will provide summaries of intervention effects for each study by calculating risk ratios (for dichotomous outcomes) or standardised mean differences (for continuous outcomes) from the data presented in the published studies or obtained from study authors.

#### Statistical analysis

We anticipate that there will be limited scope for meta-analysis because of the range of different outcomes measured across the reasonably small number of existing mobile technology intervention trials. However, where studies have used the same type of intervention and MED, with the same outcome measure, we will use Stata v11.0 [31] to pool the results of randomised controlled trials using a random-effects meta-analysis, with standardised mean differences for continuous outcomes and risk ratios for binary outcomes, and calculate 95% confidence intervals and two sided P values for each outcome. In studies where the effects of clustering have not been taken into account, we will adjust the standard deviations for the design effect, using intra-class coefficients, if they are provided in the study reports, or alternatively using external estimates obtained from similar studies[32]. Heterogeneity between the studies in effect measures will be assessed using both the χ^2 ^test and the *I*^2 ^statistic. We will consider an *I*^2 ^value greater than 50% indicative of substantial heterogeneity. We will conduct sensitivity analyses based on study quality (risk of bias; level of participant drop-out) in order to investigate possible sources of heterogeneity). We will use stratified meta-analyses to explore heterogeneity in effect estimates according to: study quality (allocation concealment and blinding in RCTs); study populations (primary versus secondary prevention for health behaviour change interventions and high versus low and middle income country settings); the logistics of intervention provision (fully automated intervention content versus content generated by healthcare professionals or other qualified personnel); and intervention content (information only versus explicit use of behaviour change theories and techniques for behaviour change interventions). We will assess evidence of publication bias using Egger's weighted regression method for continuous outcomes and Begg's rank correlation test for dichotomous outcomes.

## Conclusion

This systematic review of M-health interventions will provide a detailed summary of the evidence for the effectiveness of mobile computing and communication technologies to improve a broad range of health and health service outcomes.

## Competing interests

The authors declare that they have no competing interests.

## Authors' contributions

PE and CF initiated and designed the study. LF, VP and LG participated in study design. GP participated in study design and drafted the manuscript. All authors read and approved the final manuscript.

## Supplementary Material

Additional file 1 **MEDLINE (Ovid) search strategy**. Search terms used to identify studies of interventions using mobile computing and communication technologies in MEDLINE (Ovid). Click here for file
